# Supplementary material for: Properties and Crystal Structure of the Cereibacter sphaeroides Photosynthetic Reaction Center with Double Amino Acid Substitution I(L177)H + F(M197)H
Source: Membranes (Basel). 2023 Jan 26;13(2):157. doi: 10.3390/membranes13020157 (PMC9964780; doi:10.3390/membranes13020157)
Supplement: Supplementary file 1 [file membranes-13-00157-s001.zip › membranes-2159709-supplementary.pdf]

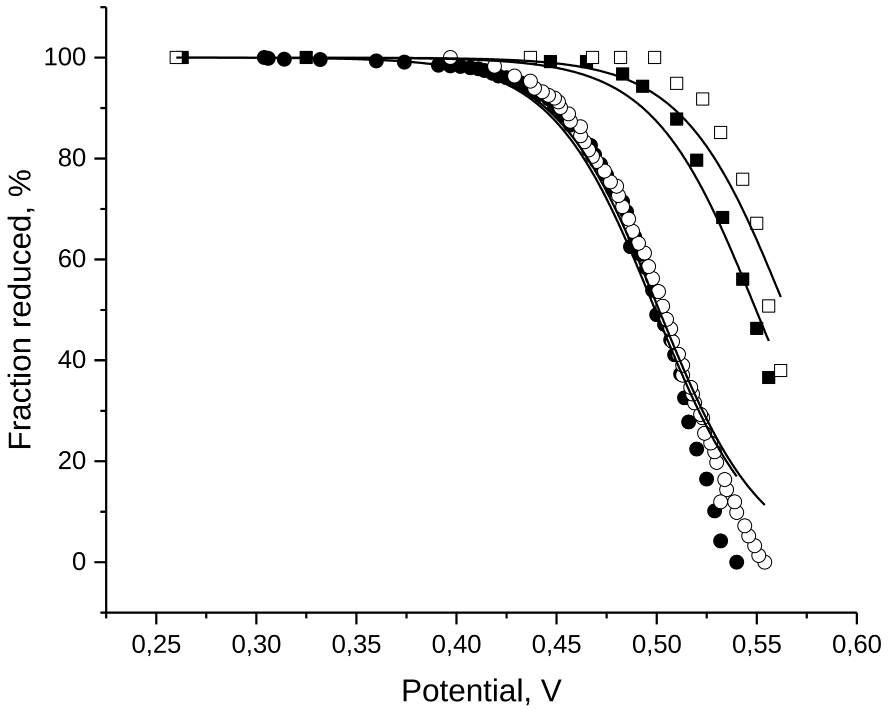

Figure S1. Redox titrations of the wild-type (●,○) and the I(L177)H+F(M197)H (■,□) RCs. The extent of reduction of the P dimer was determined by measuring the amplitude of the P Q_y band (at 865 nm in WT and 854 nm in mutant) simultaneously with monitoring the ambient redox potential. The close symbols represent normalized data from oxidative titrations, and the open symbols represent normalized data from subsequent reductive titrations performed on the same sample. The lines show the fits to the Nernst equation (n = 1). According to the data, the values of the P/P^+ midpoint potential for the wild-type and double mutant RCs are approximately 500±1 and 555±10, respectively.

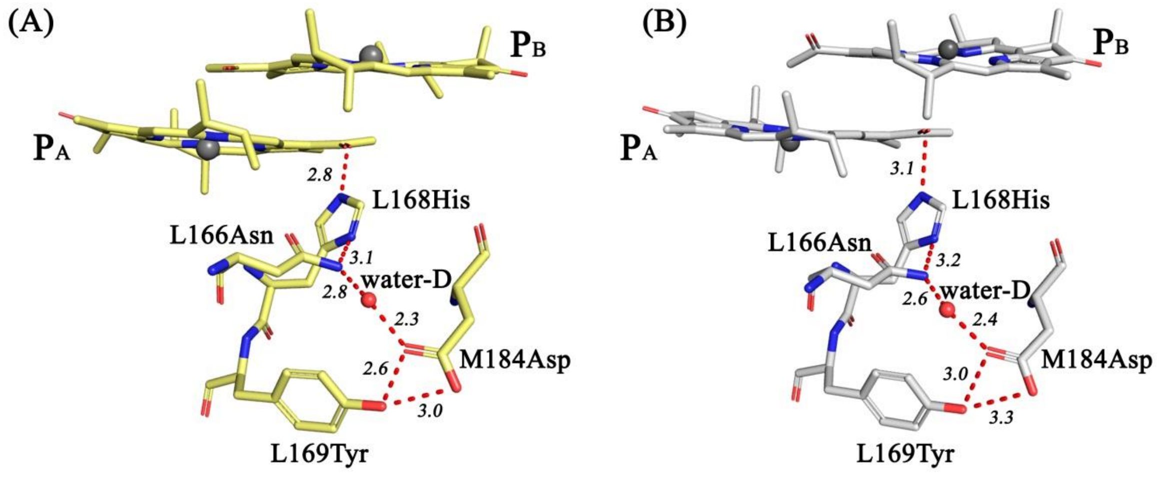

Figure S2. The hydrogen-bonding networks in the proximity of the His L168 in the wild-type RC (A) and the RC I(L177)H + F(M197)H (B). All distances are shown in Å.
